# CCL-DTI: contributing the contrastive loss in drug–target interaction prediction

**DOI:** 10.1186/s12859-024-05671-3

**Published:** 2024-01-30

**Authors:** Alireza Dehghan, Karim Abbasi, Parvin Razzaghi, Hossein Banadkuki, Sajjad Gharaghani

**Affiliations:** 1 Department of Bioinformatics, Kish International Campus, University of Tehran, Kish, 1417614411 Iran; 2 Laboratory of System Biology, Bioinformatics and Artificial Intelligence in Medicine (LBB&AI), Faculty of Mathematics and Computer Science, Kharazmi University, Tehran, 1417614411 Iran; 3 Department of Computer Science and Information Technology, Institute for Advanced Studies in Basic Sciences (IASBS), Zanjan, 4513766731 Iran; 4 Laboratory of Bioinformatics and Drug Design (LBD), Institute of Biochemistry and Biophysics, University of Tehran, Tehran, 1417614411 Iran

**Keywords:** Drug discovery, Multimodal deep learning, Drug–target interaction, Contrastive loss function

## Abstract

**Background:**

The Drug–Target Interaction (DTI) prediction uses a drug molecule and a protein sequence as inputs to predict the binding affinity value. In recent years, deep learning-based models have gotten more attention. These methods have two modules: the feature extraction module and the task prediction module. In most deep learning-based approaches, a simple task prediction loss (i.e., categorical cross entropy for the classification task and mean squared error for the regression task) is used to learn the model. In machine learning, contrastive-based loss functions are developed to learn more discriminative feature space. In a deep learning-based model, extracting more discriminative feature space leads to performance improvement for the task prediction module.

**Results:**

In this paper, we have used multimodal knowledge as input and proposed an attention-based fusion technique to combine this knowledge. Also, we investigate how utilizing contrastive loss function along the task prediction loss could help the approach to learn a more powerful model. Four contrastive loss functions are considered: (1) max-margin contrastive loss function, (2) triplet loss function, (3) Multi-class N-pair Loss Objective, and (4) NT-Xent loss function. The proposed model is evaluated using four well-known datasets: Wang et al. dataset, Luo's dataset, Davis, and KIBA datasets.

**Conclusions:**

Accordingly, after reviewing the state-of-the-art methods, we developed a multimodal feature extraction network by combining protein sequences and drug molecules, along with protein–protein interaction networks and drug–drug interaction networks. The results show it performs significantly better than the comparable state-of-the-art approaches.

## Introduction

Drug–target interactions (DTI) prediction is vital to drug discovery, as it helps to identify potential interactions between drugs and targets [1–4]. In particular, DTI prediction focuses on identifying whether the specific proteins interact with a drug compound or not [5]. Additionally, it offers guidance on drug repurposing, multi-drug pharmacology, drug resistance, and side effect prediction [6, 7]. The traditional biomedical measurement of DTI through in vitro experiments is considered reliable, but it is costly, time-consuming, and inefficient, particularly when dealing with large-scale datasets [8–11]. However, computational methods for DTI prediction have been receiving increased attention [12–14]. The current techniques for predicting DTI can be categorized into three distinct groups: ligand-based [15], docking-based [16], and machine learning-based approaches [10].

In recent years, DTI prediction has gotten more attention [17–19]. The introduced methods could be divided into two categories: feature-based methods and similarity-based methods. Zhang and Xie [20] introduced a DTI model based on non-negative matrix factorization. They introduced a new L_2,1 regularization term to guarantee the sparsity of the feature matrices derived through non-negative matrix factorization. They have proved that the obtained solution converges to the KKT point. Feature-based methods include two main modules: the feature extraction module and the task prediction module. In the feature extraction module, raw sequences of protein and drug molecules should be mapped to discriminative feature spaces. Ozturk et al. [21] introduced a DeepDTA model, which utilizes two 1D convolution networks to learn feature space for drugs and proteins. Then, the drug and protein feature vectors are concatenated to be fed into the task prediction model. Karimi et al. [22] introduced a semi-supervised method that first learns two sequence-to-sequence models to learn an initial representation of a drug-target pair. Then, it is used as an initializer for the RNN-CNN network as a feature extractor of the pair. Li et al. [13] introduced a co-contrastive learning-based method for DTI prediction to learn more discriminative representation for drug target pairs. To do so, they have utilized inhomogeneous graph representation. Qian et al. [23] introduced an approach using the drug chemical text information and the drug 2D structure image as input. Moreover, they have utilized a bi-directional multi-head cross-attentional module to encode drug and target interaction features. Zhang et al. [24] have used a transformer based model containing graph-based layers to extract features from drug molecules and a convolutional network to extract features from protein sequences. Yazdani-Jahromi et al. [25] introduced a method called AttentionSiteDTI. They treat the drug–target complex as a sentence to identify the effective protein binding sites that contribute to the drug–target interaction. In the task prediction module, the goal is to take the feature descriptor of the drug-target pair to produce the task label as output. Many approaches use a simple multilayer perceptron as a task prediction network. Tayebi et al. [26] introduced UnbiasedDTI, which focuses on the imbalance issue of the active/inactive classes in DTI. They have introduced an ensemble of deep-learning models to cope with this issue. He et al. [27] extract cross-view knowledge, including the sequence and network views for drugs and targets. They have utilized contrastive loss to learn better feature vectors for drugs and targets. To do so, they have defined auxiliary contrastive losses, including (1) contrasting similar and dissimilar drug feature vectors in sequence view, (2) contrasting similar and dissimilar drug feature vectors in network view, (3) contrasting similar and dissimilar target feature vectors in sequence view and (4) contrasting similar and dissimilar target feature vector in network views. Li et al. [13] introduced a new Supervised Graph Co-contrastive Learning for Drug–Target Interaction Prediction called SGCL-DTI. Thay have defined two graphs: topological graph and semantic graph where in these graphs, nodes are the drug-target pairs. Then, supervised contrastive loss is defined over these feature representaions. Zhnag et al. [28] introduced a new method in DTI called MRBDTA. They have introduced a modified version of the transformer encoder with skip connections. Also, they have introduced an effective approach to better encode the knowledge of the interaction site between drug and protein. In [29], a graph convolutional network (GCN) extracts features from proteins and drugs. To do so, they have extract protein 2D graph by using protein contact matrix and its physicochemical properties of residues. To extract the intra-molecular interactions, they have utilized cross-attention layers. Then, inter- and intra-molecular features are fused to feed into the MLP network.

**Fig. 1:**
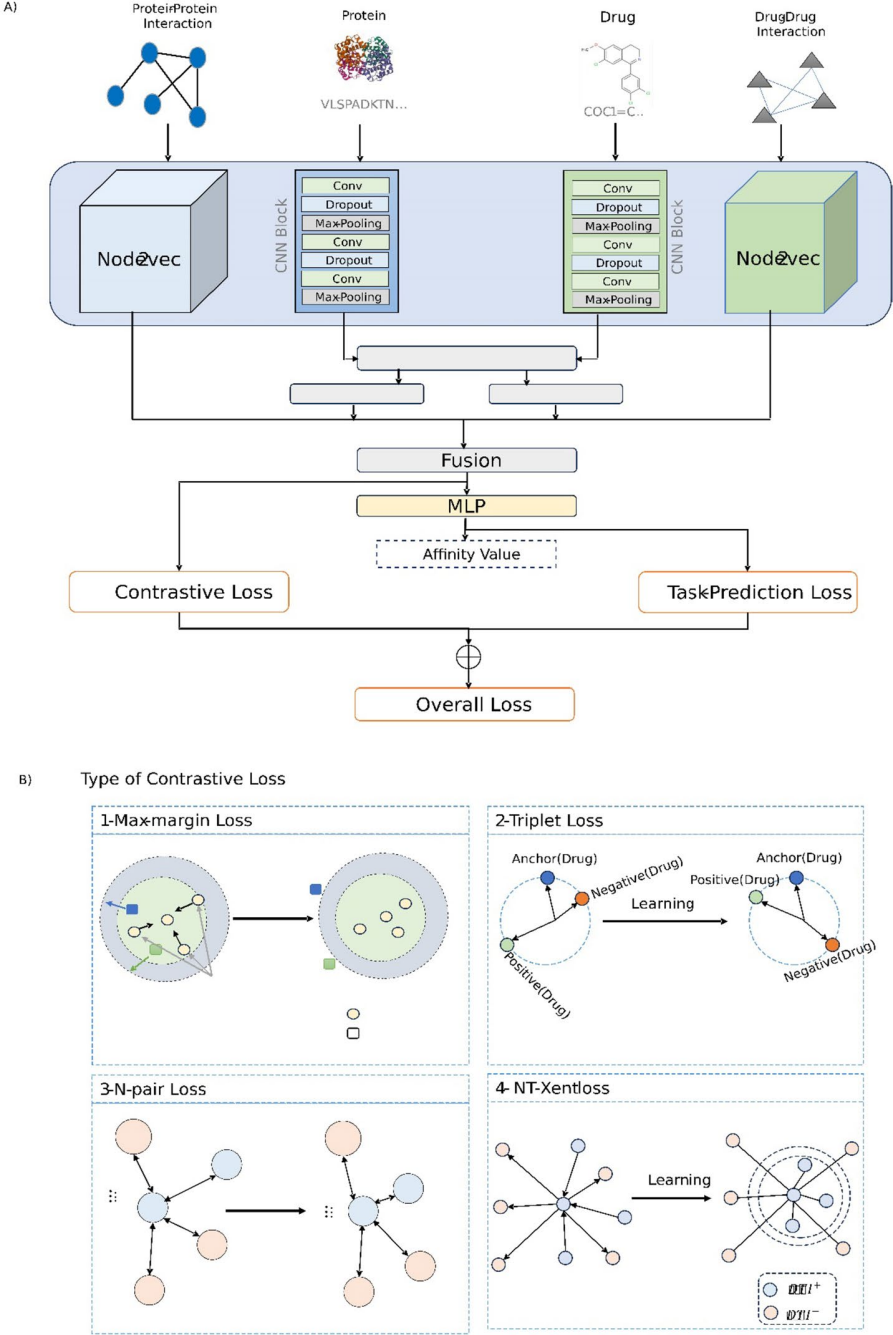
An overview of the proposed approach

In this paper, the research question is, "How do the different contrastive loss functions impact the drug target interaction prediction model's performance?". To investigate this research question, we present a new approach with two stages: (1) the first stage considers architecture to extract appropriate features for proteins and drugs, and (2) the second stage, a combinational loss function that includes task prediction loss and contrastive loss. For the feature extractor network, the first stage, we have utilized multimodal knowledge as input, including the drug molecule, protein sequences, protein–protein interaction networks, and drug–drug interaction networks. To extract features from the protein–protein interaction graph and drug–drug interaction graph, we have used the Node2vec network. To extract features from protein sequences and drug molecules, the 1D-convolution neural networks are used. We have used the two-sided attention mechanism to fuse the knowledge of these different modalities. Finally, the outputs of these networks are concatenated and fed into a multi-layered perceptron (MLP) to predict the affinity value. To recap, this comprehensive approach allows for a more complete understanding of the complex relationship between drugs and their targets, potentially leading to more accurate predictions. To investigate the effect of different contrastive loss functions, we have considered four important contrastive loss functions: (1) triplet loss function, (2) max-margin contrastive loss function, (3) Multi-class N-pair Loss Objective, and (4) NT-Xent loss function. The overall architecture of the proposed model is shown in Fig. 1. In the proposed approach, we have two loss functions to train the model: (1) the task prediction loss and (2) the contrastive loss function. In the training step, the model is first trained by the contrastive loss function, and then we train the model based on the prediction loss function. Next, this procedure is repeated until convergence is happened. It should be noted that providing data for contrastive loss functions is important. Each input data includes two drug-target pairs in the max-margin contrastive loss function. In the triplet loss function, we need three drug-target pairs, including anchor, positive, and negative. In Multi-class N-pair Loss Objective and NT-Xent loss functions, each input sample contains N drug-target pairs.

We evaluated the proposed approach on four well-known datasets: Wang et al. [30], Luo's dataset [31], KIBA [32], and Davis [33]. The results show significant improvements compared to state-of-the-art approaches and the base approach. It confirms that learning the discriminative feature space of the drug-target pair helps the task prediction model to predict the affinity value accurately.

To recap, the contributions of this paper are as follows:

1. We have utilized a multimodal feature extractor network. It means that the proposed method leverages various sources of information beyond considering the drug molecule and protein sequences. Specifically, it takes into account the drug–drug interaction network and protein–protein interaction network, providing a broader perspective on the interplay between drugs and their targets.
2. We have proposed an attention-based fusion technique to combine the knowledge of the different modalities. To do so, we have utilized a two-sided attention mechanism.
3. We have used four powerful contrastive loss functions along the task prediction loss to learn more discriminative feature space.
4. We have done huge experiments to compare the contrastive loss functions in learning more discriminative feature space.
5. The results confirm the effectiveness of using contrastive loss functions along the task prediction loss function.

This paper is organized as follows: first, the problem formulation is given, and then, the proposed method is explained in detail. Next, the evaluations of the method’s performance are presented. Finally, the paper highlights its effectiveness and suggests potential areas for further research.

## Proposed method

In this section, we have given the details of the proposed method. The main contributions of this paper are to (1) fuse the multimodal knowledge using the attention-based module and (2) evaluate how different contrastive loss functions could impact drug–target interaction prediction. To do so, first, we have given the problem formulation. Next, the model's architecture is given, and finally, we have defined the different contrastive loss functions.

### Problem formulation

Given $\{ \left( {d^{\left( i \right)} ,p^{\left( i \right)} } \right),l^{\left( i \right)} )\}$ where $\left( {d^{\left( i \right)} ,p^{\left( i \right)} } \right)$ is a drug-target pair and $l^{\left( i \right)}$ is its corresponding affinity value or activity label (active or inactive). A drug, $d^{\left( i \right)}$, is shown by Simplified Molecular Input Line Entry System (SMILES) sequence, and ith protein is shown by amino-acid sequence. SMILES is a language to translate a three-dimensional chemical molecule into a string of symbols. The main goal is to design a system that takes the drug-target pair as input and predicts affinity value as output.

### Model architecture

The architecture of the proposed approach is presented in this section. It consists of three subnetworks: protein feature encoder, drug feature encoder, and affinity value predictor (as task predictor). This paper uses the protein–protein interaction, drug–drug interaction networks, and protein sequence and drug molecule as input. PPI network is fed into the node2vec to extract feature vectors, and the same procedure is done for DDI. For extracting features from drug molecules and protein sequences, two 1D CNN networks are utilized. To combine the knowledge of the drugs and proteins, we have utilized the attention mechanism. In this case, we have utilized a two-sided attention mechanism. First, the drug features are considered as a query, and protein features are considered as key and value. Conceptually, it weights each local substructure of the protein sequence contributing to the drug features. Then, the protein features are considered as a query, and drug features are considered as key and value. It determines the contribution of each local substructure of the drug molecule in updating protein features. Finally, the drug molecule features, drug–drug interaction graph features, protein sequence features, and protein–protein interaction graph features are concatenated and fed into the task prediction network. The task prediction network is a multilayer perceptron. A schematic view of the model architecture is shown in Fig. 1. In the following, the whole feature encoder is shown by $N_{E}$. To recap, the network $N_{E}$, takes drug SMILES, protein sequence, PPI, and DDI as input and returns the feature descriptor as output.

### Contrastive loss function

In this section, the different types of loss functions are introduced and defined. In metric learning, metrics are learned to measure the similarity or dissimilarity between objects. Contrastive loss functions were introduced specifically for metric learning, aiming to optimize the parameters of these functions using deep neural networks. The resulting model can capture complex relationships between features and generate high-quality representations by embedding data points into a lower-dimensional space through deep neural networks. Ultimately, the objective is to create a model that renders a pair of examples with the same label more similar than a pair of examples with different labels. In this paper, four types of contrastive loss functions are used as auxiliary loss functions to learn a better model, and finally, in the experimental section, we evaluate these loss functions and explain how they perform.

#### Max-margin contrastive loss

The max-margin contrastive loss function was initially introduced by Hadsell et al. [34]. This loss function aims to maximize the distance between the pair of samples that belong to different classes. The max-margin contrastive loss function is defined as follows:

1 $${\mathcal{L}}_{{max{ - }margin}} \left( {z_{i} ,z_{j} } \right) = 1_{{l^{\left( i \right)} = l^{\left( j \right)} }} \left\| {z_{i} - z_{j} } \right\|_{2}^{2} + 1_{{l^{\left( i \right)} \ne l^{\left( j \right)} }} {\text{max}}\left( {0,m - \left\| {z_{i} - z_{j} } \right\|_{2}^{2} } \right)$$

where $z_{i}$ denotes the output of the feature encoder network for the ith sample $z_{i} = N_{E} \left( {d^{\left( i \right)} ,p^{\left( i \right)} } \right)$. This loss function for samples with similar labels minimizes the Euclidean distance between their corresponding feature vector. The Euclidean distance between the dissimilar samples (with different class labels) should be greater than the predefined margin threshold *m*.

#### Triplet loss function

The triplet loss function was first introduced by Weinberger [35], then it was used as a loss function by Facenet to train the deep neural network [36]. This loss function operates on triplets. Given $\left\{ {\left( {d,p} \right),\left( {d,p} \right)^{ + } ,\left( {d,p} \right)^{ - } } \right\}$ as a triplet include an anchor sample shown by $\left( {d,p} \right)$, positive sample shown by $\left( {d,p} \right)^{ + }$ which has a same class label with anchor sample, and negative sample shown by $\left( {d,p} \right)^{ - }$ which has a different class label with an anchor sample. This loss function is defined as follows:

2 $${\mathcal{L}}_{Triplet} \left( {z_{i} ,z_{j} ,z_{k} } \right) = {\text{max}}\left( {0,\left\| {z_{i} - z_{j} } \right\|_{2}^{2} - \left\| {z_{i} - z_{k} } \right\|_{2}^{2} + m} \right)$$

where $m$ shows the margin, this loss function aims to minimize the distance between the feature embedding of the anchor and positive samples and maximize the distance between the anchor and negative samples.

One of the most important disadvantages of the triplet loss function is that only one negative example in each sample is considered, and the relation of that negative example with other negative samples (especially from different negative classes) is not considered. This problem leads to slow convergence for the triplet loss function.

#### Multi-class N-pair loss objective

This loss function is introduced by Sohn [37] for the first time. Given $\left\{ {\left( {d,p} \right),\left( {d,p} \right)^{ + } ,\left( {d,p} \right)^{ - ,1} ,\left( {d,p} \right)^{ - ,2} , \ldots , \left( {d,p} \right)^{ - ,N - 1} } \right\}$ as (N + 1)-tuple of the training samples where $\left( {d,p} \right)$ is the anchor sample. Also, $\left( {d,p} \right)^{ + }$ denotes the positive samples to $\left( {d,p} \right)$ and $\left( {d,p} \right)^{ - ,i}$ shows ith negative sample to $\left( {d,p} \right)$. Hence, the N-pair loss function is defined as follows:

3 $${\mathcal{L}}_{NT - Xent} \left( {\left\{ {\left( {d,p} \right),\left( {d,p} \right)^{ + } ,\left( {d,p} \right)^{ - ,1} ,\left( {d,p} \right)^{ - ,2} , \ldots , \left( {d,p} \right)^{ - ,N - 1} } \right\}} \right) = {\text{log}}\left( {1 + \mathop \sum \limits_{k = 1}^{N - 1} \exp \left( {z^{T} z_{k} - z^{T} z^{ + } } \right)} \right)$$

where $z$ and $z^{ + }$ denotes the output of the feature encoder network for anchor and positive sample. Also, $z_{k}$ denotes the output of the feature encoder network for $k{\text{th}}$ negative sample. As it is clear, it is the generalized version of the triplet loss function, which considers more than one negative example. It is shown that when *N* is set to two, it is identical to the triple loss function. One of the most important disadvantages of minimizing Eq. (3) loss function is that generating a batch is expensive. For each batch sample, we need (N + 1)-tuple. Sohn [37] considered this issue by introducing a new approach to generating batches.

#### NT-Xent loss function

NT-Xent was first introduced by Chen et al. [38] for normalized temperature-scaled cross-entropy loss. This loss function is similar to multi-class N-pair loss with the difference that a new variable called temperature is introduced to consider the scale of the similarity values. Chen et al. [38] introduced the NT-Xent loss function for semi-supervised learning. Khosla et al. [39] modified this loss function for a supervised setting, which is defined as follows:

4 $${\mathcal{L}}_{NT - Xent - supervised} \left( {z_{i} } \right) = \frac{ - 1}{{2N_{{l^{\left( i \right)} }} - 1}}\mathop \sum \limits_{j \in P\left( i \right)}^{ } log\frac{{{\text{exp}}\left( {z_{i} z_{j} /\tau } \right)}}{{\mathop \sum \nolimits_{k \in A\left( i \right)}^{ } {\text{exp}}\left( {z_{i} z_{k} /\tau } \right)}}$$

where $\tau$ denotes the temperature parameter, one of the most important findings about the temperature is that it could help the approach to learn a better model from hard samples. Chen et al. [38] showed that the value of the temperature parameter is dependent on batch sizes and the number of training epochs. Also, $A\left( i \right)$ shows all samples in the batch distinct from $i$, and $P\left( i \right)$ is the set of all samples in the batch that they have the same label with $i{\text{th}}$ sample.

The proposed approach uses these contrastive loss functions along the task-specific loss function to learn a better model. In other words, the overall loss function of the proposed model is defined as follows:

5 $${\mathcal{L}}_{overall} = {\mathcal{L}}_{contrastive} + {\mathcal{L}}_{task prediction}$$

where ${\mathcal{L}}_{contrastive}$ is one of four introduced contrastive loss functions and ${\mathcal{L}}_{task prediction}$ is the task-specific loss function. If the affinity value is continuous, the task-specific loss function is the mean-squared error, and if it is discrete, the task-specific loss function is the categorical cross-entropy. It should be noted that all introduced contrastive loss functions are supervised, and they utilize the corresponding discrete class labels. Hence, we need to convert the continuous labels to discrete ones for the regression task to use in contrastive loss functions.

## Experiments

In this section, the experimental results are given. Four well-known datasets are used to evaluate the proposed method: Wang et al. [30], Luo's dataset [31], KIBA [32], and Davis[33]. In the following, we first introduce datasets; next, the experimental setting is explained. After that, evaluation metrics are introduced, and finally, the obtained results are given and analyzed.

### Datasets

Wang et al. dataset: there are six heterogeneous networks included in Wang et al. [30]: (1) drug–drug interactions network, (2) protein–protein interactions network, (3) drug–protein interaction network, (4) drug–disease associations, (5) protein–disease associations, and (6) drug side effects associations. The drug–target interaction network contains 1923 edges extracted from Drugbank Version 3.0 [40–43]. In this paper, we have used only the drug–drug interactions network, protein–protein interactions network, and drug–protein interaction network.

KIBA dataset: it is a well-known DTI dataset containing 117,657 interaction pairs. These pairs are from 2,068 unique drugs and 229 unique target proteins. The affinity value for each pair is measured by the KIBA score, which is an integration of IC_50_, K_(i)_, and K_(d)_ scores [44]. KIBA is a large dataset, and there are many varieties in the unique number of drugs and proteins. For the KIBA dataset, similar to [44], the threshold value is set to 12.1 and it is used to convert the predicted continuous values into binary values.

Davis dataset: it is another well-known DTI dataset containing 25,772 interaction pairs. These pairs are from 68 unique drugs and 442 unique target proteins. In this dataset, the binding affinity is measured by $k_{d}$ value. To have a more stable learned model, the $k_{d}$ value should be transformed into the log space as follows:

6 $$pK_{d} = - log_{10} \left( {\frac{{K_{d} }}{{10^{9} }}} \right)$$

This study also converts the predicted continuous values into binary values by applying thresholds. Similar to [44], the selected threshold for Davis is set to 7.

Luo Dataset: This dataset is a heterogeneous graph [31] in which there are four different types of nodes: proteins (1512 nodes), drugs (708 nodes), side-effects (4192 nodes), and diseases (5603 nodes). Also, there are eight types of edges (i.e., interaction), including drug–protein interaction (1923 edges), protein–protein interaction (7363 edges), drug–drug interaction (10,036 edges), drug–disease interaction (199,214 edges), drug–side effect interaction (80,164 nodes) and protein–disease interaction (1,596,745 edges).

### Evaluation metrics

We must select important evaluation metrics for regression and classification tasks in the proposed approach. In the regression task, we choose two metrics to evaluate the performance: (1) The Concordance Index (CI) measures the degree of ranking agreement between the predicted and ground truth values. (2) The R^2^ measure provides insight into the percentage of the dependent variable variance that the model can explain. For the classification task, we have considered five evaluation measures: (1) Recall, which measures the ratio of positive samples that are correctly classified from all positive samples; (2) Precision, which considers how good the classifier is at avoiding false alarms.; (3) Accuracy measures the ratio of correctly classified samples; (4) Area under the ROC curve (AUC-ROC), and (5) Area under the precision-recall curve (AUC-PR).

### Results

**Fig. 2:**
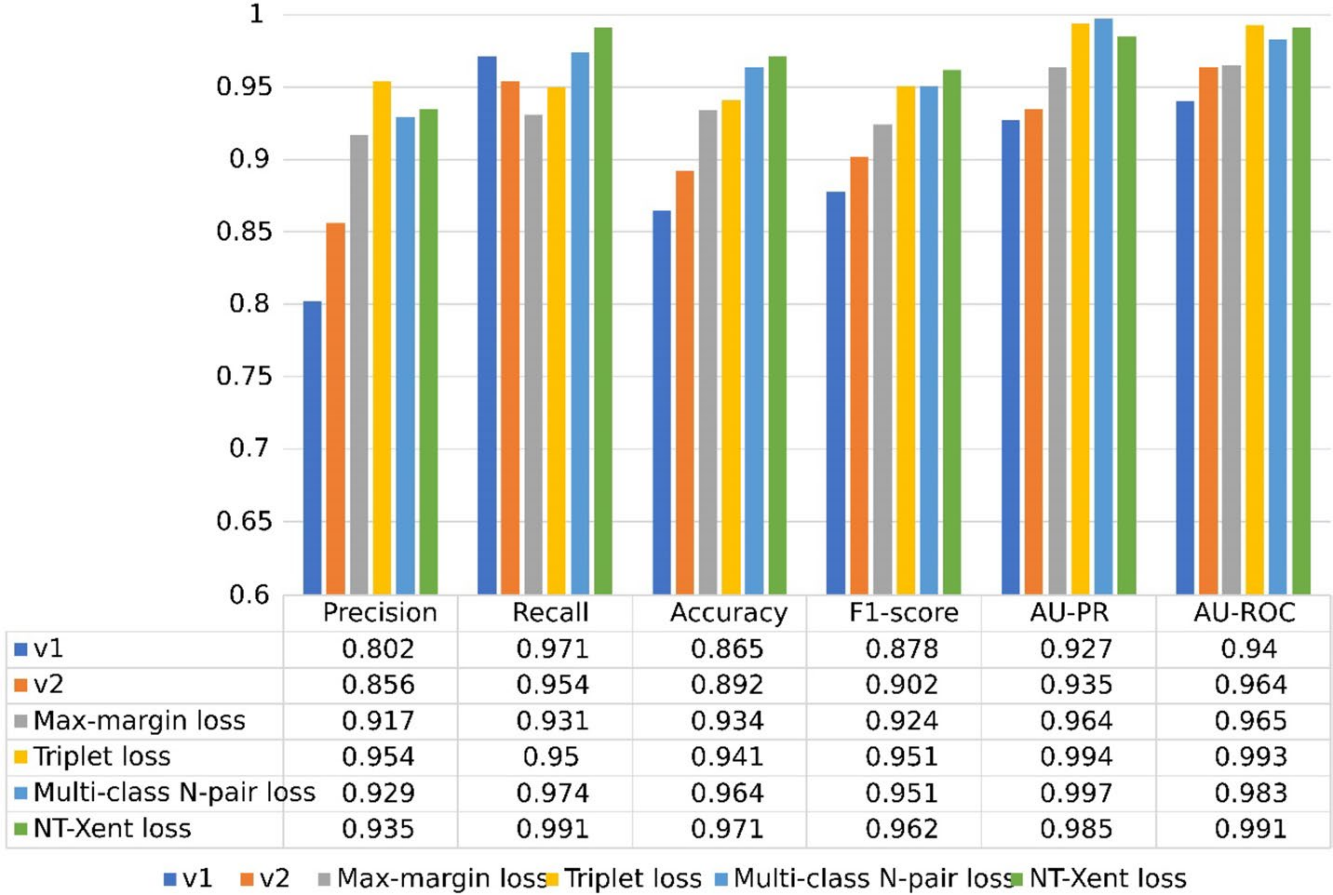
The ablation study on the Wang et al. dataset

This section presents the results obtained on four datasets. First, the results of the ablation study by Wang et al. are shown in Fig. 2. The ablation study evaluates six versions of the proposed method: (1) v1: the network is trained without attention-based fusion and contrastive loss functions. In this case, a simple concatenation is used to fuse the multimodal knowledge. (2) v2: in this case, the architecture is the same as the proposed model, and the contrastive loss is not used. In the following models, the architecture is the same as the proposed architecture, and the effect of the different contrastive loss functions is evaluated. (3) Triplet loss: The overall loss function is equal to the sum of the task prediction loss and the triplet loss; (4) Max-margin loss: The loss function for this case is the sum of task prediction loss and max-margin loss; (5) Multi-class N-pair loss: the overall loss function is the sum of task prediction loss and Multi-class N-pair loss, and (6) NT-Xent loss: the overall loss function sums the task prediction loss and the NT-Xent loss. As is shown in the proposed approach, the contrastive loss function is set to one of the four mentioned losses, and the obtained results are reported.

**Fig. 3:**
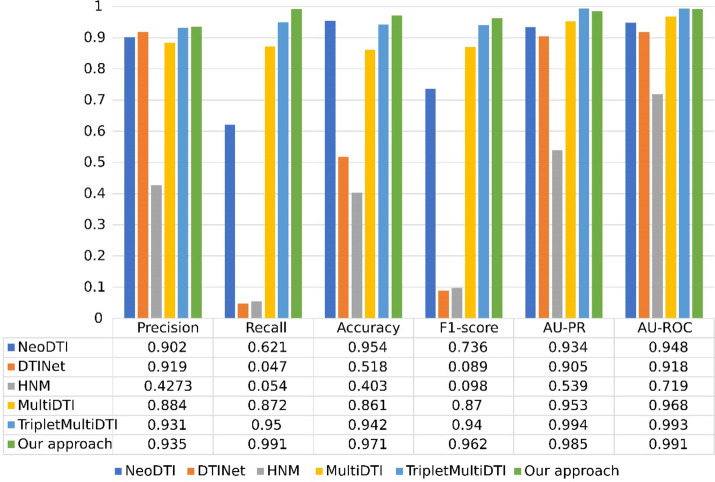
Results obtained from Wang et al. dataset and comparisons with state-of-the-art approaches

A comparison of the proposed method with state-of-the-art methods is shown in Fig. 3. Our approach is compared to five state-of-the-art approaches, including MultiDTI [45], DTINet [31], NeoDTI [46], HNM [30], and TripletMultiDTI [3]. As shown in four metrics, the proposed method performs better than the other comparable approaches. It confirms that utilizing an appropriate contrastive loss function along the task prediction loss helps the model learn more discriminative feature space, leading to increased performance.

The obtained results on Luo's dataset are given in Table 1. As shown in accuracy and AUROC, the proposed method performs better than the other approaches. Also, our approach achieves a comparable performance in other metrics compared to the best state-of-the-art approaches. It should be noted that MOVE utilizes a contrastive loss function [47], too, and our approach could improve three out of six measures over this approach.

**Table 1:** Luo's dataset results and comparison with state-of-the-art methods

	Acc	Precision	Recall	F1-measure	AUROC	AUPR
*State-of-the-art*						
DTINet	0.524	**1.000**	0.048	0.090	0.914	0.932
MultiDTI	0.536	0.649	0.538	0.417	0.822	0.842
NeoDTI	0.877	0.884	0.868	**0.876**	0.944	0.952
SGCL-DTI	–	–	–	–	0.977	**0.977**
MOVE	0.876	0.858	**0.904**	**0.876**	0.950	0.943
*Our approach*						
Task prediction loss	0.728	0.994	0.597	0.746	0.854	0.861
Triplet loss	0.794	0.964	0.634	0.765	0.919	0.921
Max-margin loss	0.741	0.999	0.619	0.764	0.897	0.918
Multi-class N-pair loss	0.859	0.814	0.869	0.841	0.967	0.958
NT-Xent loss	**0.878**	0.847	0.873	0.860	**0.978**	0.964

The bold ones mean that it is the best performance of that measure among the comparable methods

Table 2 shows the results of the proposed method in the Davis dataset. For the Davis and KIBA datasets, we have compared the proposed method with the following approaches: KronRLS [48], SimBoost [44], DeepDTA [21], DeepCDA [1], SimCNN-DTA [49], GraphDTA [50], NerLTR-DTA [51], and TripletMultiDTI [3]. As shown, the obtained results are reported for the four different contrastive loss functions and a model with only task prediction loss. The obtained results are significantly better than TripletMultiDTI when the NT-Xent loss function is used as a contrastive loss function [3]. To statistically evaluate the proposed method, we have used the paired t-test. In this test, the null hypothesis states that there is no significant difference between the proposed approach and the comparing methods. Based on the reported p-values in Table 2, we reject the null hypothesize with a p-value lower than 30% for all state-of-the-art methods except the TripletMultiDTI approach.

**Table 2:** Davis dataset results and comparison with state-of-the-art methods

	Approaches	CI measure	AU-PR	*p* value
State-of-the-art	KronRLS	0.871 ± 0.0008	0.661 ± 0.010	2.91 × 10^–1^
	SimBoost	0.872 ± 0.002	0.709 ± 0.008	2.41 × 10^–1^
	DeepDTA	0.878 ± 0.004	0.714 ± 0.010	2.54 × 10^–1^
	DeepCDA	0.891 ± 0.003	0.739 ± 0.006	2.65 × 10^–1^
	GraphDTA	0.893	–	–
	NerLTR-DTA	0.936	–	–
	SimCNN-DTA	0.855 ± 0.0027	0.657 ± 0.0076	2.54 × 10^–1^
	TripletMultiDTI	0.901 ± 0.001	0.902 ± 0.007	8.06 × 10^–1^
Our approach	Task prediction loss	0.874 ± 0.003	0.821 ± 0.004	6.39 × 10^–2^
	Triplet loss	0.901 ± 0.001	**0.902 ± 0.007**	8.06 × 10^–1^
	Max-margin loss	0.901 ± 0.004	0.842 ± 0.007	5.48 × 10^–2^
	Multi-class N-pair loss	0.928 ± 0.006	0.873 ± 0.004	2.83 × 10^–1^
	NT-Xent loss	**0.945 ± 0.005**	0.879 ± 0.004	

The bold ones mean that it is the best performance of that measure among the comparable methods

Table 3 shows the results obtained by applying the proposed method to the KIBA dataset. As presented, the proposed method effectively increases the performance with respect to the comparable approaches. It should be noted that the task is a regression task for the Davis and KIBA datasets. It means that the model predicts a continuous value. This leads us to utilize CI measures for both of these datasets. Also, we have converted the continuous affinity value to a binary label by thresholding like [1, 21, 44]. The CI measure and AUPR are increased by 2.9% and 5.6% over the best state-of-the-art method. In other words, it means the model learns a strong model by utilizing both the appropriate contrastive loss function and the prediction loss function. Based on the reported p-values in Table 3, we reject the null hypothesis with a p-value lower than 20% for most state-of-the-art methods.

**Table 3:** KIBA dataset results and comparison with state-of-the-art methods

	Approaches	CI measure	AU-PR	*p* value
State-of-the-art	KronRLS	0.782 ± 0.0009	0.635 ± 0.004	1.82 × 10^–1^
	SimBoost	0.836 ± 0.001	0.760 ± 0.003	1.34 × 10^–1^
	DeepDTA	0.863 ± 0.002	0.788 ± 0.004	1.72 × 10^–1^
	DeepCDA	0.889 ± 0.002	0.812 ± 0.005	2.48 × 10^–1^
	GraphDTA	0.891	–	
	NerLTR-DTA	0.893	–	
	SimCNN-DTA	0.821 ± 0.0011	0.721 ± 0.0018	1.61 × 10^–1^
	TripletMultiDTI	0.895 ± 0.003	0.839 ± 0.007	2.00 × 10^–1^
Our approach	Task prediction loss	0.882 ± 0.005	0.798 ± 0.002	2.42 × 10^–1^
	Triplet loss	0.895 ± 0.003	0.839 ± 0.007	2.00 × 10^–1^
	Max-margin loss	0.901 ± 0.010	0.870 ± 0.009	3.89 × 10^–2^
	Multi-class N-pair loss	**0.921** ± 0.006	0.881 ± 0.004	3.74 × 10^–1^
	NT-Xent loss	**0.924 ± 0.003**	**0.896 ± 0.001**	

The bold ones mean that it is the best performance of that measure among the comparable methods

## Conclusion

This paper focuses on this research question: "How contrastive loss function along the task prediction loss could help the approach to learn a more discriminative model?". We have selected four important contrastive loss functions to investigate and used them as auxiliary loss functions. However, we believe that a feature extraction network may be beneficial in learning a strong model. Accordingly, after reviewing the state-of-the-art methods, we developed a multimodal feature extraction network by combining protein sequences and drug molecules, along with protein–protein interaction networks and drug–drug interaction networks. To fuse the multimodal knowledge, we have proposed to use an attention-based fusion technique.

One of the advantages of the proposed method, which leads to performance improvement, is that it utilizes a powerful loss function. The loss function guides the optimization process during the backpropagation. Hence, using powerful loss functions leads to an improvement in the performance and the generalization capabilities of trained models. The loss function in most DTI approaches is based on the error between the predicted outputs and the ground truth labels without considering the representation vector of the drug-target pair. As a result of this work, we introduce a novel loss function that combines the task prediction loss with a contrastive loss function.

To evaluate the proposed method, it is applied to four well-known datasets: Wang et al., Luo's dataset, Davis, and KIBA datasets. A huge experiment is done to show the effectiveness of the proposed method. Based on the results obtained, the proposed method could improve the performance.

One of the limitations of the proposed method is the computational complexity. In Multi-class N-pair Loss and NT-Xent loss functions, each batch sample needs (N + 1)-tuple, which is practically intractable. Although, we have utilized an introduced approach by Sohn [32] to generate batches. Still, it needs more computing power. The other limitation is finding the best strategy to generate batches. In future work, providing more informative batches for DTI will be considered.

In recent years, ncRNAs are recognized as a new class of drug targets due to its effectiveness evidence in gene expression and disease progression [52, 53]. In future work, by providing protein-disease and ncRNA-disease graphs as additional inputs, we can modify the approach to predict small molecule-ncRNA associations.

## Data Availability

The sample data and code for CCL-DTI: https://github.com/dehghan1401/CCL-DTI.

## Abbreviations

DTI: Drug–target interactions
PPI: Protein–protein interaction
DDI: Drug–drug interaction
CNN: Convolutional neural network
SMILES: Simplified molecular-input line-entry system
CI: Concordance Index
AUC-PR: Area under the precision-recall curve
AUC-ROC: Area under ROC curve
